# Prospective assessment of the accuracy of ASGE and ESGE guidelines for choledocholithiasis

**DOI:** 10.1055/a-2089-0344

**Published:** 2023-06-21

**Authors:** Andy Silva-Santisteban, Ishani Shah, Madhuri Chandnani, Vaibhav Wadhwa, Leo Tsai, Abraham F. Bezuidenhout, Tyler M. Berzin, Douglas Pleskow, Mandeep Sawhney

**Affiliations:** 1 Div. of Gastroenterology, Beth Israel Deaconess Medical Center, Boston, United States; 2 Department of Medicine, Harvard Medical School, Boston, United States; 3 1859 Department of Radiology, Beth Israel Deaconess Medical Center, Boston, United States; 4 Department of Radiology, Harvard Medical School, Boston, United States

## Abstract

**Background and study aims** American Society of Gastrointestinal Endoscopy (ASGE) and European Society of Gastrointestinal Endoscopy (ESGE) guidelines recommend categorizing patients by risk for choledocholithiasis to determine management. The goal of our study was to compare the accuracy of criteria proposed in these guidelines.

**Patients and methods** All patients with suspected choledocholithiasis at our institution were prospectively identified. Based upon initial test results, patients were categorized as low, intermediate, and high risk for choledocholithiasis per ASGE 2010 and 2019, and ESGE criteria. Patients were followed until 30 days post-discharge. Results of endoscopic retrograde cholangiography (ERCP), endoscopic ultrasound, and magnetic resonance cholangiopancreatography were used as criteria standard for choledocholithiasis. The accuracy of each criterion for choledocholithiasis was computed.

**Results** During the study period, 359 consecutive patients with suspected choledocholithiasis were identified, of whom 225 had choledocholithiasis. Median patient age was 69 years and 55.3% were women. ESGE criteria categorized 47.9% as high-risk, lower than ASGE 2010 (62.7%, *P* <0.01), and 2019 criteria (54.6%, *P* =0.07). In high-risk patients, choledocholithiasis was noted in 83.1% for ESGE criteria, similar for ASGE 2019 (81.6%, *P* =0.7) and 2010 criteria (79.1%, *P* =0.3). The percentage of patients who underwent unnecessary ERCP was 8.1% per ESGE criteria, lower than ASGE 2010 (13.1%, *P* =0.03), but similar to 2019 criteria (10%, *P* =0.4). No difference in accuracy for choledocholithiasis was noted among the three criteria. No 30-day readmissions for choledocholithiasis were noted in the low-risk category.

**Conclusions** ESGE and ASGE guidelines have similar accuracy for diagnosis of choledocholithiasis. However, ESGE criteria result in more patients needing additional testing, but also a smaller proportion of patients undergoing unnecessary ERCP.

## Introduction

Gallstone-related disease accounts for over 1.5 million hospital visits per year in the United States alone 1 . This is the second most common cause of hospitalization and 30-day readmissions among gastrointestinal diseases. Up to 20% of patients with gallstones also have choledocholithiasis 2 . All patients with choledocholithiasis, regardless of symptoms or abnormal blood tests, are advised to undergo endoscopic retrograde cholangiopancreatography (ERCP) for stone extraction 3 . While ERCP is highly effective in managing choledocholithiasis, it is associated with an up to 10% risk of adverse events 4 . Based on results of blood tests and initial imaging studies, the American Society of Gastrointestinal Endoscopy (ASGE) and European Society of Gastrointestinal Endoscopy (ESGE) have published guidelines categorizing patients as high, intermediate, or low risk for choledocholithiasis 3 5 6 . These guidelines prioritize minimizing unnecessary ERCPs in patients without choledocholithiasis and have proposed different thresholds for when patients should be taken directly for ERCP. The ASGE 2010 guidelines suggested that patients with >50% likelihood of choledocholithiasis should undergo ERCP without additional testing 6 . However, the ASGE 2019 guidelines raised the threshold for direct ERCP 5 . The ESGE guidelines are the most selective and recommend proceeding directly to ERCP only in cases of acute cholangitis or when choledocholithiasis are confirmed on initial imaging studies 3 . A result of raising the threshold for direct ERCP is a higher proportion of patients having to undergo additional testing, which may result in a delayed or missed diagnosis of choledocholithiasis.

Several studies have validated the accuracy of ASGE 2019 and the ESGE choledocholithiasis guidelines 7 8 9 10 11 . All studies to our knowledge are retrospective. They are limited by the possibility of selection bias based upon how patients were identified for inclusion. Some studies only include patients who eventually underwent ERCP 8 10 . Others selected only those patients who were scheduled to undergo cholecystectomy or were assigned diagnostic codes for gallstone-related disease 9 10 11 . Our goal was to conduct a prospective study to compare the accuracy of ASGE 2010, ASGE 2019, and ESGE choledocholithiasis guidelines in a broad category of patients presenting with prespecified criteria that were indicative of possible choledocholithiasis.

## Patients and methods

### Patient selection, study design and data collection

All patients with suspected choledocholithiasis seen at our institution from 2021 to 2022 were prospectively identified. Inclusion criteria for the study was suspicion for choledocholithiasis, defined as the presence of epigastric or right upper quadrant abdominal pain associated with any one of the following: abnormal liver enzymes, bile duct dilation, or suspected bile duct stone on initial imaging done for pain evaluation. Patients who underwent either abdominal ultrasound or computed tomography (CT) scan as initial imaging test were included. Patients who underwent endoscopic ultrasound (EUS), magnetic resonance cholangiopancreatography (MRCP) or ERCP without undergoing abdominal ultrasound or CT scan were excluded, as were patients for whom full clinical information was unavailable. Definitions of ASGE 2010, ASGE 2019, and ESGE criteria for risk of choledocholithiasis are given in Table 1 . Based upon the results of initial clinical presentation, blood tests and imaging, patients were categorizing as having low, intermediate, and high risk probability for choledocholithiasis per these criteria. The study was designed as an observational cohort and the actual decisions regarding patient management were left to the discretion of treating physicians. Generally, patients at high risk for choledocholithiasis proceeded directly to ERCP, those categorized as intermediate risk underwent EUS or MRCP, and those categorized as low risk did not need further evaluation. Study patients were followed until hospital discharge to ascertain final outcome regarding presence or absence of choledocholithiasis. Hospital records were also searched after 30 days of study patient discharge to assess for readmission related to choledocholithiasis. The criteria standard (gold standard) for choledocholithiasis was presence or absence of stones on ERCP, EUS, intraoperative cholangiogram, or MRCP. Patient characteristics, laboratory results, imaging reports, and results of all interventions were abstracted and stored in a Redcap database.

**Table:** **Table 1** Summary of ASGE 2010, ASGE 2019, and ESGE criteria for choledocholithiasis

Probability	ASGE 2010	ASGE 2019	ESGE
High	CBD stone on US ^1^ Clinical ascending cholangitis ^1^ Bilirubin >4 mg/dL ^1^ Dilated CBD on US ^2^ Bilirubin level 1.8–4 mg/dL ^2^	CBD stone on US/cross-sectional imaging or clinical ascending cholangitis or, total bilirubin >4 mg/dL and dilated CBD on US/ cross-sectional imaging	Features of cholangitis or CBD stones identified on US
Intermediate	Abnormal LFTs other than bilirubin Age >55 years Clinical gallstone pancreatitis	Abnormal LFTs or, age >55 years or, dilated CBD on US/cross- sectional imaging	Abnormal LFTs and / or CBD dilatation on US
Low	No predictors present	No predictors present	Normal LFTs and US
CBD, common bile duct; US, ultrasound; LFT, liver function test; ASGE, American Society of Gastrointestinal Endoscopy; ESGE, European Society for Gastrointestinal Endoscopy. ASGE 2010 high probability: 1) presence of any ^1^ (very strong predictors); 2) presence of both ^2^ (strong predictors).			

We used the following definitions for study variables: Bile duct dilation was defined as duct >6 mm for those with gallbladder, and >8 mm for post-cholecystectomy patients. Acute cholecystitis was defined by presence of compatible gallbladder inflammation on imaging. Gallstone pancreatitis was defined as having acute pancreatitis, and gallstones, without any other cause of acute pancreatitis. Acute pancreatitis was defined by the presence of at least two of the following: characteristic upper abdominal pain, amylase or lipase >3 times upper limit normal, or imaging findings consistent with acute pancreatitis 12 . Acute cholangitis was defined per 2018 Tokyo criteria 13 .

### Statistical analysis

We evaluated all outcomes for normality. Categorical variables were presented as proportions and continuous variables as median with interquartile range (IQR), which was reported in parenthesis. Hypothesis testing was performed using a Pearson chi-square test for categorical variables and Wilcoxon rank sum test for continuous variables. A z-statistic was used to compare difference between proportions. We used a 2X2 table to calculate diagnostic performance characteristics of accuracy, sensitivity, specificity, positive predictive value (PPV) and negative predictive value (NPV) and corresponding 95% confidence intervals for low, intermediate, and high-risk for choledocholithiasis categories for ASGE 2010, ASGE 2019 and ESGE criteria. The accuracy of the ASGE 2010, ASGE 2019, and ESGE criteria for choledocholithiasis was also estimated using receiver operating characteristic curves – area under the ROC curve (ROC-AUC). Parameters used were calculated by a non-parametric estimator obtaining most appropriate sensitivity and specificity for given cut-off values. The ROC-AUC and 95% confidence intervals (CIs) were obtained with a non-parametric estimator. An AUC of 1.0 was defined as perfect accuracy while an AUC of 0.5 as lack of accuracy. We then performed multivariate logistic regression to study the association between choledocholithiasis and patient demographic characteristics, laboratory tests, initial imaging findings, ascending cholangitis, and gallstone pancreatitis. Stepwise logistic regression was used to obtain a model-of-best-fit for predicting likelihood of choledocholithiasis.

## Sample size calculation

To determine the number of patients needed for our study, based upon literature review we predicted that 96% of patients in the ESGE high-risk category and 90% of patients in the ASGE 2019 high-risk category would have choledocholithiasis. To detect this difference with 80% power and 95% CI, we computed a sample size of 280 patients. We then assumed that 50% of patients would have incomplete data or not undergo definitive testing for choledocholithiasis. Based upon these assumptions, we estimated that we would have to identify 450 patients with suspected choledocholithiasis for our study.

## Results

### Study population characteristics

**Fig. 1:**
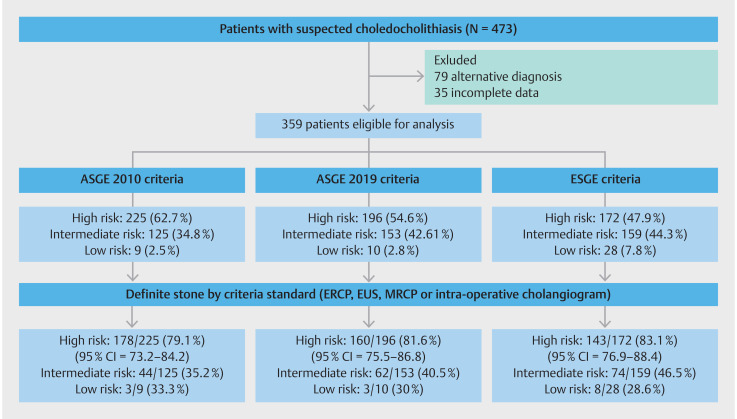
Flowchart of study subjects.

During the study period from 2020 to 2021, 473 consecutive patients with suspected choledocholithiasis were prospectively identified at our institution. In 79 patients, other explanation for clinical presentation were noted by treating physicians and, therefore, these patients did not undergo definitive testing for choledocholithiasis with either EUS, MRCP or ERCP and were excluded ( Fig. 1 ). An additional 35 patients were excluded because they were transferred to our institution and complete clinical data regarding initial presentation were unavailable. Compete data including final adjudication of choledocholithiasis per results of either EUS, MRCP or ERCP were available for 359 patients, and these patients constituted the study cohort. The median age of study patients was 69 years (interquartile range 55 to 79 years); 56% of patients were women and 69.1% were White. The initial imaging study was abdominal ultrasound in 214 patients (59.6%) and CT scan in 145 patients (40.4%). Of the patients who underwent CT scan, 99 (68.2%) were given intravenous contrast. Imaging revealed presence of a gallbladder in 269 patients (74.9%) and cholelithiasis in 189 patients (52.6%). On imaging, dilation of the common bile duct was noted in 231 patients (64.3%).

In addition, acute cholangitis was diagnosed in 85 patients (23.68%), acute cholecystitis in 45 patients (12.53%), and gallstone pancreatitis in 45 patients (12.53%). Of the 359 patients, 225 met study criteria standard for diagnosis of choledocholithiasis, and the remaining 134 patients were designated as not having choledocholithiasis. Of the 225 patients with confirmed choledocholithiasis, stones were initially noted on ERCP in 163 patients (72.44%), MRCP in 58 patients (25.8%), EUS in three patients (1.3%), and intraoperative cholangiogram in one patient (0.4%) ( Table 2 ).

**Table:** **Table 2** Baseline characteristics of study patients.

Characteristics	Total (N=359)
Age years (IQR)	69 (55–79)
Sex	
Male	158 (44.01%)
Female	201 (55.99%)
Total bilirubin=1.8–4mg/dl	113 (31.48)
Total bilirubin >4 mg/dl	73 (20.33%)
Abnormal LFTs (any abnormality)	321 (89.42%)
Abnormal LFTs (≥2 times normal)	263 (73.26%)
Abnormal LFTs (≥3 times normal)	242 (67.41%)
CBD dilation on imaging	231 (64.35%)
CBD dilation and Total Bilirubin 1.8–4 mg/dL	69 (19.22%)
CBD dilation and Total Bilirubin >4 mg/dL	58 (16.16%)
Acute cholecystitis	45 (12.53%)
Ascending cholangitis	85 (23.68%)
Gallstone pancreatitis	45 (12.53%)
Initial imaging	
Ultrasound	214 (59.61%)
CT scan	145 (40.39%)
Met criteria standard for choledocholithiasis	225 (100%)
ERCP	163 (72.44%)
MRCP	58 (25.77%)
EUS	3 (1.33%)
IOC	1 (0.44%)
IQR, interquartile range; LFT, liver function test; US, ultrasound, CT, computed tomography; ERCP, endoscopic retrograde cholangiopancreatography, MRCP, magnetic resonance cholangiopancreatography; EUS, endoscopic ultrasound, IOC, intraoperative cholangiogram.	

### Diagnostic performance of ASGE 2010, ASGE 2019 and ESGE guideline criteria

The 359 study patients were categorized into high, intermediate, and low risk for choledocholithiasis per ASGE 2010, ASGE 2019 and ESGE criteria ( Fig. 1 ). ESGE criteria categorized 47.9% of all patients as high risk for choledocholithiasis, and this was lower than that for ASGE 2010 criteria (62.7%, *P* <0.01), and ASGE 2019 criteria (54.6%, *P* =0.07). ESGE criteria categorized 46.5% of patients as intermediate risk for choledocholithiasis, and this was higher than that for ASGE 2010 criteria (34.8%, *P* <0.01), but not ASGE 2019 criteria (42.6%, *P* =0.7). ESGE categorized 7.8% of patients as low risk for choledocholithiasis, and this was higher than that for ASGE 2010 criteria (2.5%, *P* <0.01) and ASGE 2019 criteria (2.8%, *P* <0.01).

Of the patients categorized as high risk for choledocholithiasis, the proportion of patients who had a stone was 83.1% per ESGE criteria, and this was similar to the proportion of patients with stones categorized as high risk by ASGE 2019 criteria (81.6%, *P* =0.7) and by ASGE 2010 criteria (79.1%, *P* =0.3). A similar trend was observed for patients categorized as intermediate risk and low risk by the three criteria ( Fig. 1 ). The proportion of patients who underwent ERCP without discovery of stones was 8.1% (29/359) per ESGE criteria, and this was lower compared with ASGE 2010 criteria (13.1%, 47/359, *P* =0.03), but statistically similar to ASGE 2019 criteria (10%, 36/359, *P* =0.4).

The test characteristics of high-, intermediate-, and low-risk categories for choledocholithiasis for ASGE 2010, ASGE 2019 and ESGE were computed ( Table 3 ). As expected, the accuracy for choledocholithiasis for the high-risk category was greater when compared to intermediate- and low-risk categories within the three criteria. However, there was no statistically significant difference between test characteristics when ASGE 2010, ASGE 2019, and ESGE criteria were compared with each other, as demonstrated by widely overlapping CIs. There was a trend toward ESGE criteria having highest specificity and PPV for choledocholithiasis, and ASGE 2010 guidelines having highest sensitivity and NPV for choledocholithiasis; however, these differences were not statistically significant, as demonstrated by widely overlapping CIs.

**Table:** **Table 3** Diagnostic characteristics of ASGE 2010, ASGE 2019, and ESGE criteria for choledocholithiasis.

Test %, (95% CI)	ASGE 2010			ASGE 2019			ESGE		
	**High risk**	**Intermediate risk**	**Low risk**	**High risk**	**Intermediate risk**	**Low risk**	**High risk**	**Intermediate risk**	**Low risk**
Accuracy	73.82 (68.94–78.29)	26.74 (22.23- 31.64)	36.49 (31.50- 41.70)	71.87 (66.91–76.46)	29.25 (24.59- 34.25)	36.21 (31.23–41.42)	69.08 (64.02- 73.83)	34.26 (29.36–39.42)	33.98 (29.09- 39.14)
Sensitivity	79.11 (73.21- 84.23)	19.11 (14.19- 24.87)	1.33 (0.28- 3.85)	71.11 (64.71- 76.94)	27.56 (21.83- 33.89)	1.33 (0.28- 3.85)	63.56 (56.90- 69.85)	32.89 (26.79- 39.45)	3.56 (1.55 6.89)
Specificity	64.93 (56.21- 72.96)	39.55 (31.22- 48.36)	95.52 (90.51- 98.34)	71.11 (64.71- 76.94)	32.09 (24.29- 40.70)	94.78 89.53- 97.87)	78.36 (70.42- 85.00)	36.57 (28.42- 45.32)	85.07 (77.89- 90.64)
PPV	79.11 (74.87- 82.80)	34.68 (28.19- 41.79)	33.33 (11.28–66.29)	81.63 (76.86–85.61)	40.52 (34.85- 46.46)	30.00 (10.13- 61.97)	83.14 (77.88–87.35)	46.54 (40.97–52.20)	28.57 (15.34–46.89)
NPV	64.93 (58.24- 71.07)	22.55 (18.96- 26.60)	36.57 (35.66- 37.50)	60.12 (54.52- 65.47)	20.87 (16.91–25.48)	36.39 (35.41–37.38)	56.15 (51.33- 60.86)	24.50 (20.32- 29.23)	34.44 (32.76- 36.16)
ASGE, American Society of Gastrointestinal Endoscopy; European Society for Gastrointestinal Endoscopy; CI, confidence interval; PPV, positive predictive value; NPV, negative predictive value.									

**Fig. 2:**
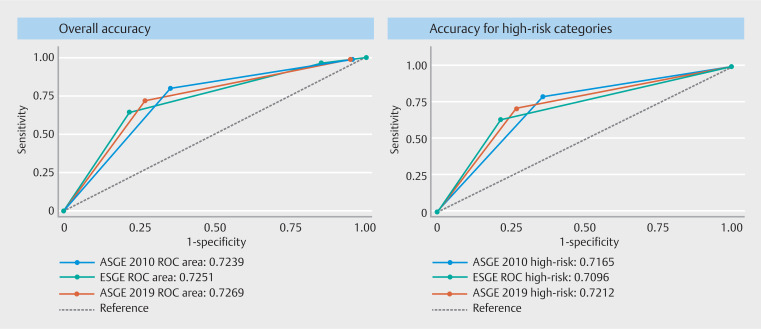
**a** Overall accuracy of ASGE 2010, ASGE 2019 and ESGE criteria for choledocholithiasis. **b** Accuracy of ASGE 2010, ESGE 2019 and ESGE high-risk categories for choledocholithiasis.

We calculated the overall accuracy of each guideline criteria using the AUC-ROC ( Fig. 2 **a** ). The overall accuracy for the diagnosis of choledocholithiasis for ASGE 2010 criteria was 0.72 (95% CI, 0.67–0.76), ASGE 2019 was 0.73 (95% CI, 0.67–0.77) and ESGE was 0.73 (95% CI, 0.67–0.77), and these differences were not statistically significant ( *P* =0.9). We also calculated the accuracy for high risk for choledocholithiasis category for each guideline criteria using the ROC-AUC ( Fig. 2 **b** ). We found that the accuracy for choledocholithiasis for the ASGE 2010 high-risk category was 0.72 (95% CI, 0.66–0.76), for ASGE 2019 was 0.72 (95% CI, 0.67–0.76), and for ESGE was 0.71 (95% CI, 0.66–0.75), and these differences were not statistically significant ( *P* =0.7).

We computed the diagnostic characteristics of each predictor variable for choledocholithiasis ( **Supplementary Table 1** ). Stones on initial imaging studies had the highest accuracy (specificity 89.4% and PPV 87.9). We then performed a univariate logistic regression to analyse the association between choledocholithiasis and predictor variables. Variables that were strong predictors of choledocholithiasis are shown in Table 4 . We then created a multivariate logistic regression model with choledocholithiasis as an outcome variable. Stepwise logistic regression was used to determine model-of-best-fit. In the final model, only presence of stones on initial imaging studies (OR=8.80, 95% CI, 4.64–16.67, *P* <0.001) and elevation of liver enzymes ≥3 times normal (OR=2.66, 95% CI, 1.61–4.40, *P* <0.001) were independent predictors of choledocholithiasis. We also performed subgroup analysis for patients who underwent abdominal ultrasound as the initial test and found no difference in the accuracy of the three guidelines.

**Table:** **Table 4** Univariate logistic regression of predictors for choledocholithiasis.

Predictors	Odds ratio	Standard error	*P* value	95% confidence interval
Age >55 years	1.77	0.43	0.02	1.09–2.87
Elevation of LFTs ≥2 times	3.06	0.75	0.00	1.88–4.95
Elevation of LFTs ≥3 times	3.32	0.78	0.00	2.10–5.26
Stone on initial imaging	8.54	2.60	0.00	4.70–15.53
CBD dilation	1.96	0.44	0.03	1.26–3.04
TB >4 mg/dL	1.97	0.56	0.01	1.12–3.46
CBD dilation + TB 1.8–4 mg/dL	2.80	0.88	0.00	1.51–5.20
CBD dilation + TB >4 mg/dL	2.32	0.76	0.10	1.22–4.42
Acute cholangitis	2.60	0.73	0.00	1.49–4.54
LFT, liver function test; CBD, common bile duct; TB, total bilirubin.				

We reviewed medical records 30 days after hospital discharge for each study patient. A total of 28 of 359 patients were defined as low risk for choledocholithiasis by any of the three criteria. A 30-day readmission for choledocholithiasis was noted in 22 patients (6.1%, 95% CI, 3.9%–9.1%). Of these, 17 were planned admissions to remove stones as previous procedures were limited by patients being on antithrombotic drugs, large stones that would not be removed, or intolerance to anesthesia. There were five unplanned admissions for biliary obstruction from choledocholithiasis. No 30-day readmission for choledocholithiasis was noted for any patients who were categorized as low risk by any of the three criteria.

## Discussion

We found no difference in overall accuracy for detection of choledocholithiasis between ASGE 2010, ASGE 2019, and ESGE criteria. The ESGE criteria categorize the smallest proportion of patients (47.9%) as high risk for choledocholithiasis, those who should proceed directly to ERCP, followed by ASGE 2019, and ASGE 2010 criteria. The ESGE criteria categorize the highest proportion of patients as intermediate risk for choledocholithiasis, those who would require further investigations with MRCP or EUS to assess for stones, followed by ASGE 2019, and ASGE 2010 criteria. The ESGE criteria result in the smallest proportion of patients undergoing ERCP where no stones were discovered (8.1%), followed by ASGE 2019, and ASGE 2010 criteria. No 30-day readmission for choledocholithiasis was noted in patients who were designated as low risk by any criteria.

The question of whether the ASGE 2019 guidelines are superior to the 2010 guidelines has not been fully settled; therefore, we included assessment of the 2010 guidelines in our study. Three prior studies that have compared these guidelines are all retrospective and differ in patients included in their analysis. Chandra et al retrospectively identified 744 patients, all of whom underwent ERCP for suspected choledocholithiasis 8 . Patients with cholecystectomy or prior biliary intervention were excluded. In this study, 36.8% of patients were categorized as high risk by 2019 ASGE guidelines, of which 82.5% had choledocholithiasis. The ASGE 2010 guidelines categorized 60.4% as high risk, of which 76.2% had stones. The overall accuracy of ASGE 2019 guideline was only 50.8%, with sensitivity of 41.5%, and specificity of 76%. No difference in PPV or NPV was noted between the two guidelines. Hasak et al identified 1098 patients with choledocholithiasis on ERCP or intraoperative cholangiogram 11 . ASGE 2019 guidelines had an accuracy of only 70.4% for choledocholithiasis, but were better than the 2010 guidelines (accuracy 60.1%). The AUC for high-risk criteria using the 2019 guidelines was 0.73 (95% CI, 0.7–0.76), which was also greater than for the 2010 guidelines 0.65 (95% CI, 0.61–0.68). Jacob et al conducted a retrospective cohort study of 265 patients with suspected choledocholithiasis, almost all of whom underwent ERCP 10 . They found that the ASGE 2010 guidelines categorized 62% patients as high risk, of whom 79% had choledocholithiasis. The ASGE 2019 criteria categorized 32% patients as high risk, of whom 83% had choledocholithiasis. While these studies differ substantially in design and their estimates of the test characteristics of the two criteria, the overall trend is consistent with our results that the 2019 ASGE are slightly better than the 2010 ASGE criteria as fewer patients are categorized high risk for choledocholithiasis, and within this category, a slightly higher proportion are found to have choledocholithiasis. Neither criterion, however, achieved high accuracy for choledocholithiasis. ASGE 2010 criteria use serum bilirubin >4 mg/dL or levels between 1.8 mg/dL and 4 mg/dL, and bile duct dilation as high-probability indicators of choledocholithiasis. These indicators were replaced in the ASGE 2019 criteria by serum bilirubin >4 mg/dL and bile duct dilation. These three variables do not differ significantly in their test characteristics for choledocholithiasis, accounting for the small incremental change in accuracy between the ASGE 2010 and 2019 criteria ( **Supplementary Table 1** ).

Two studies have compared the ASGE criteria with the ESGE criteria for choledocholithiasis. Wangchuk et al conducted a retrospective cohort of 280 patients and found similar accuracy between the ASGE 2019 high-risk group (AUC 0.75) and the ESGE high-risk group (0.74) for choledocholithiasis 7 . In this study, choledocholithiasis was found in a higher proportion of patients categorized as high-risk by the ASGE 2019 criteria (75.5%) than those categorized as high risk by the ESGE criteria (66%). Jagtap et al retrospectively assessed 1042 patients with suspected choledocholithiasis who were scheduled to undergo cholecystectomy 9 . Their results contradicted those of Wangchuk et al as they found ESGE guidelines better identified patients with choledocholithiasis. ASGE 2019 criteria categorized 22.1% as high risk, of which 89.5% were found to have choledocholithiasis, while the ESGE criteria categorized 20.4% as high risk, of which 96.2% were found to have choledocholithiasis. We found no significant difference between the overall accuracy of the ESGE and the ASGE 2019 criteria. Our estimates of the accuracy for choledocholithiasis are in line with those reported by Wangchuk et al, but lower than those reported by Jagtap et al. We prospectively identified all patients with suspected choledocholithiasis in our study and used results of blood tests and initial imaging studies obtained at the time of presentation to designate a patient’s choledocholithiasis risk category. Retrospective studies including that by Jagtap et al likely used variables obtained at different timepoints to compute the choledocholithiasis risk category, possible explaining difference in our results. Categorizing a patient’s risk for choledocholithiasis is most helpful in determining further patient management when done at the time of presentation; therefore, we contend that our results are more pertinent to clinical practice. We enrolled a wide range of patients with suspected choledocholithiasis in our study, 40% of whom underwent CT scan of the abdomen as their initial diagnostic study. No difference in the test characteristics of ASGE and ESGE criteria were noted when either results of ultrasound or of CT scan were used for computations.

Results of the aforementioned studies along with ours show that the ESGE criteria are the most restrictive in categorizing patients as high risk for choledocholithiasis. Only those patients with acute cholangitis or stones documented on initial imaging studies were recommended to proceed directly with ERCP. This comes at the expense of designating a larger proportion of patients as intermediate risk for choledocholithiasis, resulting in an increase in EUS and MRCP procedures. At institutions with expertise and ready availability of EUS and MRCP, this appears to be a reasonable strategy, as the ESGE criteria result in the fewest number of patients undergoing unnecessary ERCP who do not have bile duct stones. However, at other institutions, this strategy may result in missed stones or a delay in diagnosis. A meta-analysis found marked variation among difference centers in their accuracy of EUS and MRCP for choledocholithiasis 14 . The sensitivity of EUS ranged from 0.8 to 1.0 and the sensitivity of MRCP ranged from 0.4 to 1.0. Furthermore, MRCP may have lower accuracy for small stones, and these may be missed at institutions that rely primarily on MRCP for assessing intermediate-risk patients 15 16 . We suggest that institutions with limited expertise and availability of EUS and MRCP may consider using the ASGE 2019 criteria for such patients. Reassuringly, none of the patients placed in the low-risk category were readmitted within 30 days for choledocholithiasis. Of note, overall 30-day readmission rates noted in our study were lower than those reported in a nationwide database 17 .

## Conclusions

In summary, we found no difference in the overall accuracy of ASGE 2010, ASGE 2019, and ESGE criteria for choledocholithiasis. The ESGE criteria were most restrictive, resulting in the greatest number of patients requiring further testing with EUS or MRCP, but the fewest number of patients undergoing unnecessary ERCP. Based on expertise and availability of EUS and MRCP, we suggest that institutions adapt criteria most suitable to their circumstances.

## Supporting information

Supplementary material Zusatzmaterial
