# Supplementary material for: Prospective assessment of the accuracy of ASGE and ESGE guidelines for choledocholithiasis
Source: Endosc Int Open. 2023 Jun 21;11(6):E599–606. doi: 10.1055/a-2089-0344 (PMC10442906; doi:10.1055/a-2089-0344)
Supplement: Supplementary file 1 — Supplementary material [file 10-1055-a-2089-0344_20900497.pdf.pdf]

Supplementary material

Table 1. Test characteristics of ASGE 2010, 2019 and ESGE criteria variable for choledocholithiasis

High-risk predictors	Performance % (95% CI)				
	Accuracy	Sensitivity	Specificity	PPV	NPV
**CBD stones on initial imaging**	65.74 (60.58 -70.64)	50.23 (43.38 - 57.07)	89.44 (83.18 - 93.97)	87.90 (81.56 - 92.27)	54.04 (50.42 - 57.62)
**CBD dilation on initial imaging**	60.45 (55.18 - 65.54)	70.51 (63.96 - 76.49)	45.07 (36.72 - 53.64)	66.23 (62.29 - 69.97)	50.00 (43.18 - 56.82)
**TB 1.8-4 mg/dl**	48.19 (42.91 - 53.49)	33.78 (27.63 - 40.36)	72.39 (64.00 - 79.76)	67.26 (59.63- 74.07	39.43 (36.14-42.82)
**TB > 4mg/dl**	48.75 (43.47 - 54.05)	24.42 (18.86 - 30.70)	85.92 (79.09 - 91.18)	72.60 (62.38 - 80.90)	42.66 (40.21 - 45.14)
**CBD dilation + TB 1.8-4 mg/dl**	50.42 (45.12 -55.71)	24.88 (19.28 -31.19)	89.44 (83.18 -93.97)	78.26 (67.91 -85.97)	43.79 (41.47 -46.15)
**CBD dilation + TB > 4mg/dl**	47.91 (42.64 -53.22)	20.28 (15.14 -26.25)	90.14 (84.01- 94.50)	75.86 (64.16 - 84.66)	42.52 (40.43 -44.65)
**Clinical ascending cholangitis**	52.09 (46.78 -57.36)	29.95 (23.94 -36.52)	85.92 (79.09 -91.18)	76.47 (67.36 - 83.66)	44.53 (41.84 - 47.25)

CBD, common bile duct; TB, total bilirubin; CI, confidence interval.

 © 2023. Thieme. All rights reserved.
